# Patient and family experiences of early cognitive rehabilitation in critical illness: A qualitative study protocol

**DOI:** 10.1111/nicc.13254

**Published:** 2025-03-04

**Authors:** Marie Oxenbøll Collet, Louise Skov Svendsen, Anne Højager Nielsen, Eva Laerkner, Ingrid Egerod

**Affiliations:** ^1^ Department of Intensive Care Copenhagen University Hospital Copenhagen Denmark; ^2^ Centre for Research in Intensive Care, CRIC Copenhagen University Hospital Copenhagen Denmark; ^3^ Department of Anesthesiology and Intensive Care Gødstrup Hospital Herning Denmark; ^4^ Department of Clinical Medicine Aarhus University Aarhus Denmark; ^5^ Department of Anesthesiology and Intensive Care Odense University Hospital Odense Denmark; ^6^ Department of Clinical Research in Anesthesiology University of Southern Denmark Odense Denmark

**Keywords:** critical care, interview, protocol, qualitative, rehabilitation

## Abstract

**Background:**

Physical, mental and cognitive impairments are known severe adverse outcomes in critically ill patients who have beenadmitted to an intensive care unit (ICU). Early rehabilitation interventions such as lighter sedation and increased mobilization have improved patient outcomes, but evidence for early cognitive rehabilitation is sparse.

**Aim:**

The study aims to describe the experiences of ICU survivors and their families regarding early cognitive stimulation as part of rehabilitation during and after ICU admission.

**Design:**

Our study has a qualitative, descriptive design using individual and dyad interviews as well as focus groups with ICU survivors and their families.

**Methods:**

Eligible participants are adult ICU survivors and families who are able to recall events during and after the ICU stay. We will use purposeful sampling in the outpatient clinic in the Department of Intensive Care at the Copenhagen University Hospital. We plan to conduct five individual patient interviews, four dyad interviews with patients and families, and two focus groups with patients. All interviews will follow a semi‐structured interview guide based on the study objectives and input from a patient and public involvement (PPI) panel. We will use the framework method for analysis and invite the PPI panel to participate in the analysis process to strengthen the findings. Written informed consent will be obtained from all participants, and data will be anonymized before analyses.

**Relevance for Clinical Practice:**

This study protocol provides a methodological framework to understand early cognitive rehabilitation from the perspectives of ICU survivors and their families. This study protocol presents one of the first studies to investigate the concept of early cognitive rehabilitation during critical illness from this perspective. The findings will facilitate a tailored approach to early rehabilitation to be investigated in further studies.

**What is known about the topic**

- Many intensive care survivors experience long‐term cognitive impairment.
- Early intensive care rehabilitation is often driven by physiotherapists.

**What this paper adds**

- How intensive care unit (ICU) survivors and their families experience early cognitive rehabilitation.
- Barriers to early cognitive rehabilitation according to ICU survivors and their families.

## 1 INTRODUCTION

The number of patients admitted to the intensive care unit (ICU) and surviving critical illness is on the rise, with about 75% alive 30 days after discharge.^1^ This trend is expected to continue as the elderly population grows and medical treatments advance.

Critical illness is a condition of one or more organ dysfunctions caused by, for example, septic shock, trauma or respiratory failure requiring close monitoring and specialized care in the ICU. Patients in the ICU receive interdisciplinary intensive care from a team of health care professionals.

With the decreasing mortality rates in the ICU, there has been a shift from preventing death to preventing long‐term physical and psychological issues and promoting long‐term recovery and well‐being in ICU survivors. Physical, mental and cognitive impairments are collectively known as post‐intensive care syndrome (PICS).^2^ Cognitive health refers to the health of the brain, whereas mental health is the psychological or emotional health. Long‐term cognitive impairment affects up to 60% of ICU survivors and can be as severe as Alzheimer's disease, posing a growing public health care challenge.^3^, ^4^

Cognitive impairment in ICU survivors is linked to increased dependence on health care and family support, rendering even basic daily activities of living a challenge. Potential modifiable risk factors for long‐term impairment in the ICU include mechanical ventilation, sepsis, sedation and length of ICU stay regardless of cognitive function prior to critical illness.^5^, ^6^, ^7^ However, no gold standard exists for measuring and quantifying cognitive function in ICU survivors and cognitive decline is under‐recognized by intensivists and rehabilitation professionals.^8^

## 2 BACKGROUND

Early mobilization, delirium prevention, daily breathing trials and wake‐up calls, sleep protocols and family engagement have been suggested as potential interventions and early rehabilitation strategies for the prevention of cognitive impairment, but the evidence is sparse.^9^, ^10^ A scoping review identified mobilization, physical rehabilitation and activities of daily living as the most commonly reported occupational therapist (OT) supported interventions to prevent cognitive impairment in the ICU.^11^ A recent feasibility randomized clinical trial (RCT) demonstrated that cognitive stimulation, upper limb training and activities of daily living initiated by the OT were feasible during the ICU stay.^12^ A non‐blinded RCT demonstrated that extensive cognitive follow‐up and training after ICU discharge can improve cognition and expedite the recovery period.^13^ Early cognitive rehabilitation in the ICU has been described not only as a cognitive stimulation task but also as an OT task such as activities of daily living interventions, for example brushing teeth, wiping the face, washing the body, dressing or eating dependent on cognition (arousal, attention, executive functioning), physical strength and physiological restrictions.^13^

Today, physical therapy assisted by physiotherapists is common in daily early rehabilitation, but occupational therapy assisted by OTs is not very well implemented in many ICUs.^11^ The evidence regarding nurse‐led interventions directed towards early cognitive stimulation in ICU patients is limited. In addition, the patient and family experience of early cognitive rehabilitation is sparse.

## 3 AIM

The study aims to describe the experiences of ICU survivors and their families regarding early cognitive stimulation as part of rehabilitation during and after ICU admission. This study will inform the development of a nurse‐led cognitive stimulation intervention. The development will be guided by the Medical Research Council (MRC) framework for developing and evaluating complex interventions.^14^ This study is supported by a comprehensive integrative review. The protocol for this is published elsewhere.^15^

### 3.1 Research question

The specific objectives of the study are:

1. To describe the ICU survivor and family experiences of early rehabilitation in and after the ICU, and to understand the implications for recovery.
2. To investigate facilitators and barriers to early rehabilitation in and after ICU according to ICU survivors and families.

## 4 DESIGN AND METHOD

### 4.1 Study design

The study has a qualitative descriptive design triangulating interview methods with ICU survivors and their families.

### 4.2 Study site and setting

The study will be conducted at the Copenhagen University Hospital, Rigshospitalet, Denmark. The hospital is a national referral hospital with 1244 beds and five ICUs: thoracic, neurological, cardiac, neonatal and mixed medical/surgical ICU with a total of 60 ICU beds. The 20‐bed mixed medical/surgical ICU will host the study. Early physical rehabilitation is facilitated by an ICU physiotherapist in collaboration with an ICU nurse and physician. Patients are mobilized to a sitting position in bed or a chair as soon as their condition allows it. Routine occupational therapy is not offered.

### 4.3 Participants

The participants in this study are ICU survivors and their family caregivers. Participants will be purposively sampled at the hosting department. Eligible participants are adult ICU survivors and family that are able to recollect their time in or after the ICU. We wish to obtain a maximum variation sample with different personal and socio‐economic backgrounds, as well as a range of medical conditions, in order to include different perspectives on the potential advantages of early rehabilitation in the ICU (Table 1). Inclusion and exclusion criteria are presented in Table 2.

**TABLE 1:** Sampling of eligible participants.

Characteristics	Patient	Family
Age		
Younger (18–50 years)		
Middle age (51–75 years)		
Older (>75 years)		
Sex		
Men		
Women		
Ethnicities		
Describe if participants consent^a^		
Admission type		
Medical		
Surgical		
ICU length of stay		
Less than 7 days		
7 days or more		
Time from ICU discharge		
Less than 3 months		
More than 3 months		

^a^ Not commonly performed in Denmark.

**TABLE 2:** Inclusion and exclusion criteria.

Inclusion criteria	Adult ICU survivor (aged ≥18 years) with at least one ICU admission
	Acute critical illness
	Family caregiver (aged ≥18 years) present during the ICU admission
Exclusion criteria	Admission due to suicide attempt
	If written consent cannot be obtained

Eligible participants will be approached by a member of the research team at the outpatient clinic, peer‐support group meetings or when in contact during other studies. The research team will inform the aftercare providers about the study. The research team will not have access to potential participants' health records, but the aftercare providers may use the patient records to identify eligible participants according to the study criteria.

The aftercare providers will give study information to eligible participants. If a patient or family member agrees to participate, their name, telephone number or email will be given to the research team. The research team will then contact the eligible participants and verbally inform them about the study and obtain verbal consent.

Written informed consent will be obtained from all participants before the interview is conducted. The consent form will be sent by email, or a paper version can be sent by regular mail at the request of the participants. The participants will be asked to complete the consent form and return it to the research team prior to the interview. At the time of the interview, the consent will be reconfirmed verbally.

### 4.4 Sample size

We wish to conduct five individual patient interviews, four dyad interviews with one patient and one family caregiver, and two focus group interviews with four to six patients. We will use the concept of information power to estimate the sample size.^16^ We have a narrow aim, dense specificity, strong dialogue, and cross‐case analysis, which helps determine the sample size.^16^

### 4.5 Data collection

We will conduct individual interviews with ICU survivors, dyad interviews with survivors and families, and focus groups with ICU survivors. The individual interviews will allow an in‐depth exploration of the participants' experiences, perceptions, and emotions regarding early rehabilitation.^17^ The dyad interviews will offer insight into the interpersonal dynamics and shared perspectives of the survivor and a family caregiver who was present in the ICU. These perspectives and experiences might reveal areas of consensus or disagreement, contributing to a more nuanced understanding of early rehabilitation. We will apply comparative analysis and investigation of how these dynamics interact.^18^ The focus groups will provide insight into how individuals construct meaning collectively by discussing early rehabilitation interventions and practices. The interaction between participants might lead to diverse perspectives, opinions, and experiences, where new ideas are fostered by group dynamic exploration of early rehabilitation.^19^

Individual and dyad interviews will be conducted either at the participants' homes or at the hospital. If a participants has any restrictions imposed by COVID‐19 or other contagious diseases, we may shift to online interviews. The focus groups will meet in a room in the hospital away from ICU.

All interviews will be conducted by the same researcher (MOC), who has experience in conducting qualitative interviews and focus group interviews. The researcher will be accompanied by a research assistant (LSS) who will observe and take notes during interviews. Additional support and training will be available from the research team as required.

Interviews will be semi‐structured, using an interview guide based on the study objectives and input from a patient and public involvement (PPI) panel. The interview guide includes time and space for the participants to recall and construct their own understanding of early cognitive rehabilitation. During the interviews, the interview guide may be adjusted as needed in response to the evolving research process. The initial interview guides can be found in the Data S1, and the final version will be made available when the results are presented.

The individual interviews are scheduled to last approximately 40–60 min. The dyad interviews are scheduled to last approximately 60–80 min and the focus groups approximately 60 min.

Interviews will be audio‐recorded, and the audio files will be saved on an encrypted and logged drive at the hospital server. Field notes made by the research assistant, including any additional comments or reflections, will be scanned and saved on the encrypted server with the audio files. At dissemination, the field notes will be anonymized. The audio files will be transcribed, and all identifiers will be removed and stored at the same location as the audio files and field notes. Audio recordings and all data will be deleted before 1 January 2027.

The COREQ (Consolidated criteria for Reporting Qualitative research) Checklist^20^ will be used when writing the manuscript for the qualitative study.

By triangulation of the three methods, the data generated might capture broader experiences and perspectives of rehabilitation of ICU survivors with potential memory loss or difficulty recalling what actually happened in the ICU.^21^, ^22^

### 4.6 Data analysis

Data will be analysed using the seven‐step framework method suggested by Gale et al.^23^ Step 1: All interviews will be transcribed verbatim. Step 2: The researchers will familiarize themselves with the data material by reading reflective notes and listening to audio recordings. Step 3: At least two researchers will deductively code the first transcripts using an a priori defined analytical framework (Table 3) but also be aware of other phenomena. Step 4: The researchers will compare codes and refine the analytic framework or matrix with input from the PPI panel. Step 5: The analytic framework will be applied to the rest of the data. This is an iterative process, and researchers will go back and forth in the process to add new categories to the framework if needed. Step 6: Data will be charted into the framework and condensed. Step 7: Interpretation of data: Researchers will explore linkages, connections and relationships in the data. Alongside this process, researchers will write analytic memos, noting down impressions and ideas to be shared with other researchers.^23^ Qualitative data management software, NVivo QRS version 14.23.1.38 (QRS International Pty Ltd., Doncaster, Victoria, Australia), will be used to support data analysis.

**TABLE 3:** Framework matrix for data analysis.

	Cognitive rehabilitation	Functional rehabilitation	Other rehabilitation
Rehabilitation in the ICU described by patient			
Rehabilitation post‐ICU described by patient			
Rehabilitation needs not met			
Rehabilitation needs met by patient			
Rehabilitation needs met by family caregiver			
Rehabilitation in the ICU described by family caregiver			
Rehabilitation post‐ICU described by family caregiver			

*Note*: If other nodes need to be included in the matrix, this can be carried out and will be described during the analyses in the dissemination of the findings.

To decrease the potential for researcher bias and strengthen the findings and implications for clinical practice, the research team will meet with the PPI panel to integrate their views into the analysis process.

### 4.7 Patient and public involvement

PPI is ensured by using an already established research panel comprised of three ICU survivors, two family members of ICU survivors, one ICU nurse, one ICU physiotherapist, one clinician not from the ICU, and two researchers. The PPI panel meetings are organized to meet every third month and support various research initiatives within the Intensive Care Research Collaboration www.cric.nu. The PPI panel uses an exploratory ‘speak out loud’ approach.^24^

Overall, all PPI members considered this research to be of value in informing early rehabilitation intervention in the ICU and in raising awareness among clinicians on this topic. PPI members agreed with several suggestions by the researcher (MOC), and they were included in the development of this study protocol. The main suggestion was to regard early cognitive rehabilitation as occupational therapy and, if necessary, to describe its components.

### 4.8 Ethical considerations

Research in intensive care settings has demonstrated the potential advantages for patients participating in qualitative studies.^25^ The design of the study is to minimize any risk or burden for the participants. If a participant encounters challenges during the interviews, such as fatigue or distress, the interview will be paused, and if needed terminated. Sufficient time will be allocated for debriefing, and the researcher will provide information about available resources of support if necessary. Interviews will be scheduled at a time and location convenient for the individual patient and family.

When the PPI panel is invited to participate in the analysis process, all data and matrices will contain only anonymized data. Ethics approval has been sought from the Ethics Committee of Capital Region, and The Capital region Data protection Agency.

All participants must consent based on a verbal and written information provided. Participation in the study is voluntary, and participants can withdraw their consent at any time.

This study was approved on the 21 October 2022 by ‘Hospital and Danish Protection Agency’. No other approvals were needed according to Danish legislation described by the Danish ethics committee (F‐22045840).

## 5 DISSEMINATION

Upon study conclusion, the data will be presented as a paper for publication in a peer‐reviewed journal and at relevant conferences, locally and internationally. During the consent process, participants will have the option to request notification of the study findings, through a summary report sent by mail or via email.

Furthermore, the data output from this study will be applied in the development of an early cognitive rehabilitation intervention for ICU patients. Recommendations derived from the study will be disseminated through academic papers and conference presentations, as well as through direct communication with policymaker groups at the local, regional and national levels during meetings. An infographic summarizing the study findings, with guidance from PPI, will be created and shared with patients, families and the broader public, including through social media channels.

## 6 RIGOUR

To ensure the rigour of this study, a process of phenomenological reduction, as described by Giorgi (1997), will be employed. This involves setting aside any preconceived knowledge about the phenomenon under investigation, in this case, early rehabilitation during an ICU stay, to truthfully capture the participants' description. It is recognized that the interviewer lacks personal experience of early rehabilitation during ICU admission. However, it is understood that the iterative process of data collection and analysis will inevitably influence the researcher's perspective. Nevertheless, the researcher will make an effort to remain focused on the participants' stories and the underlying meanings within their narratives, without preconceived judgement. The research team consists of a primary investigator, two senior researchers and one professor in intensive care, all with 20–40 years of intensive care nursing experience. The research assistant has 5 years of nursing experience. The team's professional values are grounded in health and nursing science, emphasizing the establishment of close relationships as the fundamental approach for engaging with individual patients.

## 7 STRENGTHS AND LIMITATIONS

The study is strengthened by the experience of the research team and the inclusion of the PPI panel in the research process. Early and in‐depth patient and public involvement is used to ensure study acceptability, relevance and output. Trustworthiness is increased by using well‐known methodology. Limitations of the study include the single‐centre approach and the relatively small sample, which is inherent to qualitative studies. Generalization is not possible, but we expect that transferability will be possible at ICUs within a similar context. Various potential sources of bias must be acknowledged, including recruitment bias, recall bias, and unconscious research bias.

## 8 IMPLICATIONS AND PERSPECTIVES

This study protocol represents one of the first studies to investigate the concept of early cognitive rehabilitation during critical illness from the perspectives of patients and families. The absence of evidence in this specific context emphasizes the significance of undertaking this type of study to create new empirical knowledge. It is recognized that findings from qualitative research are context‐specific and may not be generalizable to different settings or populations. Nevertheless, the ambition is that these findings will provide a comprehensive and detailed understanding of the early rehabilitation phenomenon in the ICU from the perspectives of ICU survivors and their families, which can inform the development of future rehabilitation interventions in clinical practice.

## AUTHOR CONTRIBUTIONS

Conceptualisation: MOC, LSS, EL, AHN and IE. Methodology: MOC, LSS, EL, AHN and IE. Resources: MOC. Writing Original Draft: MOC. Review and Editing: MOC, LSS, EL, AHN and IE. Visualisation: MOC, LSS, EL, AHN and IE. Supervision: EL, AHN and IE. Funding acquisition: MOC.

## FUNDING INFORMATION

The study is funded by the Novo Nordisk Foundation.

## PATIENT CONSENT STATEMENT

All patients and families will freely and voluntarily give consent to take part in this study. The consent is given based on the verbal and written information provided and the understanding that they are medically and physically qualified to take part in the study.

## Supporting information


Data S1.

## Data Availability

Data sharing not applicable to this article as no datasets were generated or analysed during the current study.
