# Supplementary material for: Patient and family experiences of early cognitive rehabilitation in critical illness: A qualitative study protocol
Source: Nurs Crit Care. 2025 Mar 4;30(2):e13254. doi: 10.1111/nicc.13254 (PMC11880795; doi:10.1111/nicc.13254)
Supplement: Supplementary file 1 — Data S1. [file NICC-30-0-s001.pdf]

Interview guide: Patient interviews

**Date**	**Recorded digitally**
**Name (interviewer)**	
**Place for interview**	

**In which region do you live?**	**Recorded in Excel**<br>(other characteristics are obtained from the patient record)
**Do you have at least one parent who was born in Denmark and who has Danish citizenship?**	
**How sick did you feel you were during your stay in the intensive care unit on a scale from 0-10? Where 0 corresponds to not critical and 10 to most critical.**	

Interview guide	
	What is your name?<br><br>How are you doing now?<br>(Do you consider yourself healthy?)<br><br>How have you been physically since discharge?<br><br>How have you been mentally since discharge?<br><br>How have you been cognitively (memory/concentration) since discharge?

Introduction to key questions:
We would like to gain insight into your thoughts and considerations regarding rehabilitation at the hospital and at home. How are things going at home with dressing, brushing teeth, eating, etc.? How did you spend your time in the intensive care unit and at the hospital?

**Key questions 1**	*Try to describe a "rehabilitation" situation in the ICU.*<br>*What happened, what did you experience, who did what?*
**Key questions 2**	Try to describe a "rehabilitation" situation in the ICU provided by the nurse.<br><br>Can you recall a "rehabilitation" situation where thinking was promoted?<br>Try to describe that situation and what the nurse did.
**Key questions 3**	Can you describe a "rehabilitation" situation right after you returned home?<br>What happened, who did what, how did you experience it?
**Key questions 4**	Can you describe if your loved ones helped or participated in rehabilitation with you during your hospital and ICU stay?<br><br>What type of rehabilitation would you have liked to receive in the ICU? Please describe the situation. What would you have liked more of?<br>(physical, mental, and cognitive)<br><br>*What actions did your loved ones take to help you feel better?*<br>*What actions did the nursing staff take to help you feel better in the ICU?*

Closing question	
	What is the best thing someone could do for you?<br>What is the best thing you can do for yourselves?

Interview guide: Dyad interview

**Date**	**Recorded digitally**
**Name (interviewer)**	
**Place for interview**	

**In which region do you live?**	**Recorded in Excel**<br>(other characteristics are obtained from the patient record)
**Do you have at least one parent who was born in Denmark and who has Danish citizenship?**	
**How sick did you feel you were during your stay in the intensive care unit on a scale from 0-10? Where 0 corresponds to not critical and 10 to most critical.**	

Interview guide	
	What is your name?<br><br>How are you now?<br><br>(Do you consider the patient to be healthy? Does the relative consider themselves to be healthy?)<br><br>How have you been physically since the hospital stay?<br><br>How have you been mentally since the hospital stay?<br><br>How have you been cognitively (memory/concentration) since the hospital stay?

Key questions

We would like to gain insight into your thoughts and considerations regarding rehabilitation at the hospital and at home. How are things going at home with getting dressed, brushing teeth, eating, etc.

How did you spend your time in the intensive care unit and at the hospital?

**Key questions 1**	*Try to describe a "rehabilitation" situation in the ICU.*<br>*What happened, what did you experience, who did what?*<br><br>*To the relative: Do you agree? Is there anything missing?*
**Key questions 2**	Try to describe a "rehabilitation" situation in the ICU provided by the nurse.<br>Is the patient able to recall a situation where "thinking" was promoted?<br>What did the nurse do?<br><br>To the relative: Do you agree? Is there anything missing?
**Key questions 3**	Can you describe a "rehabilitation" situation right after you (the patient) returned home?<br>What happened, who did what, how did you experience it?<br><br>To the one who is not answering the most: Did you experience the same?
**Key questions 4**	To the relative: Can you describe the actions you took regarding rehabilitation during the hospital and ICU stay?<br><br>What rehabilitation would you have liked the patient to have received in the ICU? – try to describe the situation.<br>(physical, mental, and cognitive)<br>Was there anything you would have liked more of?

**Closing question**	What is the best thing someone could do for you?<br>What is the best thing you can do for yourselves?

Interview guide: Focus group interview

**Date**	**Recorded digitally**
**Name (interviewer)**	
**Place for interview**	

**In which region do you live?**	**Recorded in Excel**<br>(other characteristics are obtained from the patient record)
**Do you have at least one parent who was born in Denmark and who has Danish citizenship?**	
**How sick did you feel you were during your stay in the intensive care unit on a scale from 0-10? Where 0 corresponds to not critical and 10 to most critical.**	

Briefing for participating in a focus group:

- Speak up, don't talk over each other. It's important to converse with each other, not just to me.
- Be honest in your responses.
- Share what you truly think; your input matters.
- Anonymity will be maintained.
- Focus on rehabilitation/training/recovery topics.

Opening question	
**Ice-breaker**	What are your names, and how long has it been since you were ill and admitted to the intensive care unit?<br><br>Has it been challenging to get to where you are today?<br><br>OR<br><br>How have you recovered after your critical illness? Do others experience the same?"

Introduction to key questions:
We would like to gain insight into your thoughts and considerations regarding rehabilitation at the hospital and at home. How are things going at home with dressing, brushing teeth, eating, etc.? How did you spend your time in the intensive care unit and at the hospital?

**Key question 1**<br><br>*Follow-up questions*	*What do you consider to be rehabilitation during intensive care admission? And at the hospital?*<br><br>*What happened, what did you experience, who did what?"*
**Key question 2**<br><br>Follow-up questions	Now I will show you some pictures and items related to possible rehabilitation. What do you think?<br><br>*Which things do you believe are meaningful for training to return to everyday life?*<br><br>*Try to describe a situation where a nurse did something similar to this.*
**Key question 3**	What about when you came home?<br><br>In terms of rehabilitation<br>What happened, who did what, how did you experience it?
**Key question 4**<br><br>Follow-up questions	Can you describe the actions your closest relatives took regarding rehabilitation during the hospital and ICU stay?<br><br>What type of rehabilitation would you have liked the patient to receive in the ICU? Please describe the situation.<br><br>*Was there anything you wish there had been more of?*

Closing question	
	What is the best thing someone could do for you?<br>What is the best thing you can do for yourselves?

Debriefing: were you uncomfortable?
